# Relationship between Personal Values, Work Experience and Nursing Competencies among Cancer Care Nurses in Malaysia

**DOI:** 10.31557/APJCP.2021.22.1.287

**Published:** 2021-01

**Authors:** Nor Aida Maskor, Mazanah Muhamad, Steven Eric Krauss, Nik Hasnaa Nik Mahmood

**Affiliations:** 1 *Professional Development and Continuing Education, Faculty of Educational Studies, Universiti Putra Malaysia, Malaysia. *; 2 *Razak Faculty of Technology and Informatics, Universiti Teknologi Malaysia, Malaysia. *

**Keywords:** Cancer care nurse, personal value, work experience, competency

## Abstract

Oncology nurses are an essential component of cancer care teams. Nurses play a vital role in ensuring that cancer patients comply with their cancer treatment. In the cancer care nursing context, competency is not merely being skilled, but also implies the characteristic of being able to perform effectively. In addition to the need for competence, nursing is a discipline rich in values including human dignity, caring, humanity, and respect for personal privacy. Research from a variety of disciplines indicates that values often influence human behaviour in professional and work settings. It is often believed, therefore, that nurse’ values and work experience influence and contribute to their work performance. Few studies have attempted to examine these relationships, particularly in the context of cancer care nursing. The purpose of this study was to determine the relationship between personal values, work experience and competency among cancer care nurses in Malaysia. Quantitative surveys were used to collect the data. A total of 845 cancer care nurses from 38 public hospitals in Peninsular Malaysia participated in the study. Descriptive statistics and Pearson Product-Moment Correlations were used to analyse the data. The findings revealed positive and significant relationships between personal values and competency (*r* = 0.59, p < 0.01) and work experience and competency (*r* = 0.11, p < 0.047). The findings support the assertion that Malaysian nurses’ values and work experience are related to performance-related competency.

## Introduction

The Malaysian population is a heterogeneous, multi-ethnic group consisting of Malays (63.1%), Chinese (24.6%), Indians (7.3%) and others (0.7%) with a total population of 28.3 million (Department of Statistics, Malaysia, 2010). Cancer is a global phenomenon and major disease whose increased prevalence over the past twenty years has made it a major health issue in Malaysia (Lim, 2002). According to the National Cancer Registry (NCR), a total of 67,792 new cases were diagnosed between 2003-2005 in Peninsular Malaysia alone (Zainal Ariffin et al., 2006). The cancer burden worldwide continues to grow, with an increasing number of new cases and deaths each year (Carlson and Bultz, 2003). As a result, cancer care is increasingly being delivered within a multidisciplinary team environment, involving a host of highly skilled professionals. Every oncology department relies on a team of highly trained radiographers, physicists, pharmacists, nurses, and support staff for everyday functioning (Tho and Wong, 2006). According to Morita et al., (2004) oncology nurses specifically are those working in cancer centres or general hospitals. 

Cancer care nurses are particularly wanting in Malaysia, as the country’s oncology patient ratio stands at 1: 650,000 (Yip et. al., 2006). Time spent by an oncology nurse with a cancer patient is more significant as compared to other clinicians (Helft et al., 2011). The oncology nurse, attending to cancer patients and their families, experiences many different relationships. The uniqueness of these relationships involves not only challenges but also rewards. As nursing is an interpersonal event, both nurse and patient engage in a dynamic relationship of mutual impact (Van Rooyen et al., 2008). Cunningham et al., (2006) reported that nurses who take care of cancer patients should have knowledge related to cancer prevention, diagnosis and treatment, psychological support, death and caring for dying patients. As an important discipline of clinical medicine, high-quality nursing contributes greatly to the outcome of different kinds of therapies for cancer patients, and nurses always play a key bridging role between doctors and patients (Gemmill et al., 2011).

Values indicate what is important, worthwhile and worth striving for. Begat et al., (2005), Fealy (2004), and McNeese-Smith and Crook (2003) all concur with the statement by the World Health Organization (2003) that to be able to “meet the challenges of their profession, nurses need to be clear about why they think and act as they do, and they need to perceive themselves as being empowered” (p. 222). This provides more evidence of the importance for nurses to have a clear understanding of what their values are. Altun (2002) stated that nurses who are not aware of their professional and personal values will have difficulty in perceiving their professional role. Nurses who deal with values effectively are more likely to be promoted and achieve personal satisfaction. Altun further mentioned that the prevailing values in nursing are: aesthetics (qualities of objects, events and persons that provide satisfaction), altruism (regard for the welfare of others), equality (having the same rights and privileges), freedom (the ability to exercise choice or action), human dignity (the inherent worth of an individual), justice (fair treatment through the upholding of moral and legal principles), and truth (faithfulness to fact or reality). Experience plays a central role in models of work performance and behaviour. In addition to knowledge, skills, and motivation, there is also evidence that work experiences can shape attitudes, values, and even personality characteristics.

Hird and Noffke (1995) mentioned that nurses know that the appropriate psychomotor skills are important but when performed without knowledge, they do not constitute nursing. At the same time, nursing knowledge of health and disease processes is of little use without the requisite nursing skills. In Malaysia and globally, the nursing profession has developed through various phases in its role as a profession of primary caregiving in health care settings (Nemie, 2009). Nurses have a variety of tasks to perform and must cope with multiple tasks often based in different parts of hospitals (Siew et al., 2011). 

The nursing workforce in Malaysia comprised almost exclusively of women (Birks et al., 2009). Nurses comprise 2–3% of the total female workforce and a large proportion of the health care workforce in Malaysia (Barnett et al., 2010). Ahmad and Oranye (2010) studied 556 registered nurses in two teaching hospitals in Malaysia and found that the nursing profession is dominated by females. A Diploma in nursing is the minimum qualification to join the nursing workforce in Malaysia (Alam and Mohammad, 2009).

Boyatzis (1982) stated that competency is a capability or ability that consists of self-image, motivation, skill and social role. He grouped competency into three clusters; (1) cognitive, (2) emotional intelligence, and (3) social intelligence. Spencer and Spencer (1993) defined competence as a broad concept of knowledge, skills, values and actions. They further mentioned that competency is used to describe the required behaviour that leads to being competent. Marrelli et al., (2009) stated that competencies are the building blocks of work performance. According to Fillion et al., (2006) as cited in Cohen et al., (2010), competencies in oncology nursing include assessing patients and families, having knowledge of health promotion and family therapy, developing skills in communication, relationship building, teamwork and problem solving, and being mature as professional leaders. Yates et al., (2007) further identified the competency standards and domains of practice for specialist breast nurses in Australia. They include: (1) supportive care, (2) collaborative care, (3) coordinated care, (4) information provision and education, and (5) clinical leadership. 

According to Tzeng (2004), nurses’ competence is an individual or personal skill which is an outcome of the professional nurse training program. Tzeng and Ketefian (2003) added that competency is comprised of a group of broad abilities and practical skills. According to Zhang et al., (2001), competencies are a set of knowledge, skills, traits, motives and attitudes. There are 10 domains of competencies; (1) professional values, (2) communication, (3) teamwork, (4) management, (5) community-oriented, (6) health promotion, (7) problem solving, (8) health care, (9) education, and (10) basic public health sciences (Regina and Maria, 2008). Arvidsson and Fridlund (2005) reported that nurses’ competence is of critical importance to clients.

According to Rosenfeld et al., (2012), there are eight most critical competencies needed by the home health care nurse manager. The competencies are; (1) aligning the performance of success, (2) clinical knowledge and skill, (3) coaching, (4) communication, (5) customer/patient focus, (6) decision making, (7) facilitating change, and (8) planning and organizing work. Larsson and Butterfield (2002) reported competencies focused on 4 aspects; (i) knowledge and concepts, (ii) assessment and referral, advocacy, (iii) ethic and risk communication, and (iv) legislation and regulation. Cleary et al., (1998) found that the most highly needed competencies were; (1) general profession technical skills, (2) assessment skills, (3) ability to work independently, (4) critical thinking, (5) interpersonal communication, (6) professional orientation, (7) case management, and (8) team-building. According to Dunn et al., (2000), competency standards for nursing practise are; (1) professional practice, (2) reflective practice, (3) enabling, (4) clinical problem solving, (5) teamwork and (6) leadership. Pai et al., (1999) study in a Taiwan medical centre found that nurses rated their areas of greatest competency as being; (1) patient care, (2) overall nursing care, (3) communication, (4) teaching, (5) management, (6) self and professional growth, and (7) research skills.

Tzeng and Ketefian (2003) identified 21 nursing competencies for staff nurses in Taiwan hospital. The competencies are; (1) complex professional technical skills, (2) specific clinical skills, (3) long term care/geriatric skills, (4) multiple skills, (5) ability to supervise, (6) general professional technical skills, (7) general clinical skills, (8) assessment skills, (9) ability to work independently, (10) critical thinking/problem solving, (11) professional orientation, (12) health care system knowledge, (13) written/verbal communication, (14) interpersonal communication, (15) case management, (16) resource management, (17) team-building/teamwork, (18) leadership, (19) delegation, (20) flexibility, and (21) coping skills. Liu et al (2009) reported seven competency inventories for registered nurses in Macao. These are (1) clinical care, (2) leadership, (3) interpersonal relationship, (4) legal/ethical practice, (5) professional development, (6) teaching-coaching and (7) critical thinking/research aptitude.

Meretoja et al., (2004) developed the Nurse Competence Scale using Benner’s Competency framework. There are 73 items of the scale and were grouped into seven categories namely; helping role, teaching-coaching, diagnostic function, managing situations, therapeutic interventions, ensuring quality, and work role. By using an exploratory qualitative research design, Connelly et al., (2003) found a total of 54 competencies of charge nurses in the clinical setting. The competencies were divided into four categories of clinical setting/technical skill, critical thinking, organizational skills, and human relation skills. There are 5 national core competencies for Malaysian nurses; (1) ethics and legal practice, (2) professional nursing and midwifery, (3) leadership and management, (4) education and research, and (5) professional personal and quality improvement (Barnett et al., 2010).

Values influence job satisfaction, motivation and commitment (Tzeng, 2002). Values also affect the way people act in their personal and professional lives (Perry, 2005). Nursing is a discipline rich in values (Rassin, 2008). He cautioned that nursing profession should not be focused on just scientific knowledge and technical skills, but also on specific human values. Central values for nurses are; (1) human dignity, (2) caring, (3) humanity, and (4) respect for personal privacy (Itzhaky et al., 2004). Hewison (2001) stated that personal values influence the way people operate in large institutions. According to Parks and Guay (2009), personal values are learned beliefs that serve as guiding principles about how individuals ought to behave. 

Personal values can reflect the nurses’ attitudes and influence professional lifestyle (Altun, 2000 and Horton et al., 2007). According to Horton et al., (2007), nurses have to understand their values in order to become good ones. Being a good nurse means that the nurse’s concern for patients is totally related to efficient, effective and attentive care that fosters patients’ well-being (Natalia and Elma, 2010). Ersoy and Altun (1998) reported 22 personal values among nurses in Turkey. They are honesty, trust, respect, independence, professional competency, humaneness, equality, justice, benevolence, rationality, altruism, economic power, sensitivity, knowledge, correctness, courage, diligence, friendship, politeness, tolerance, adaptivity and happiness. 

Experience plays a fundamental role in the development of nursing practice and value (Arbon, 2004 and Radwin, 1998). Khomeiran et al., (2006) study on 27 nurses found that they learn from their own experiences. Pitayavatanachai (2005) reported that working experience has a significant effect on nursing competency. Meretoja et al., (2004) studied competency of 593 registered nurses working in emergency/outpatient or intensive care units or in operation rooms in Finland with more than 10 years of work experience in different university hospital work setting. They found that the length of work experience had a positive correlation with the self-assessed overall level of nursing competency. Humpel and Caputi (2001) reported that there is a significant relation between emotional competency and year of experience in mental health nursing. Salonen et al., (2007) found that there is a positive correlation between current work experiences with self-assessed competency level among 235 registered nurses working in intensive and emergency settings. According to Okomato et al., (2008) among public health nurses in Japan, the more their experience, the higher the competency level achieved. There is also a significant relationship between nursing experience and competence level among 101 critical care nurses in the US (O’leary, 2012). 

*Study aims*

To examine relationship between personal values, work experience and nursing competencies among cancer care nurses in Malaysia. The objectives of this study to; (i) identify the profile of cancer care nurses in Malaysia, (ii) identify cancer care nurse competencies, (iii) identify cancer care nurse’s personal values, (iv) identify the relationship between personal value and competency, and (v) identify the relationship between work experience and competency.

## Materials and Methods

The cross-sectional study was conducted at 38 public hospitals in Peninsular Malaysia. The survey instrument was developed from various resources including literature review, qualitative focus group data and related established instruments. The questionnaire included items for competency (personal, interpersonal. and technical) and personal values using a scale ranging from 1 to 5 (strongly disagree to strongly agree). A demographic section of the questionnaire was included to obtain cancer care nurses’ social demographic profile and a number of years their working in cancer care setting. Cancer care nurses were given a questionnaire that they were asked to fill independently. Respondents required 20 – 30 minutes to complete the questionnaire.

Multistage cluster sampling technique was used to collect the data. In this study, the term cancer care nurses referred to any nurses who have working experience in caring for cancer patients. 845 respondents participated in this study from 38 public hospitals in Peninsular Malaysia.

The questionnaire was distributed to all cancer care nurses through the Matron (nurse supervisor). A brief instruction was given to the Matron regarding the questionnaire. The Matron distributed the questionnaire to respondents. The cancer care nurses participated voluntarily and anonymously in this study.

Data were entered and analyzed using the Statistical Package for Social Sciences (SPSS) version 19.0. Descriptive statistics and Pearson Product-Moment Correlations were used to analyze the data.

The questionnaire was pilot-tested with a convenience sample of 27 registered nurses in three selected hospitals. Cronbach alpha coefficients of this questionnaire ranged from 0.770 to 0.892. According to George and Mallery (2003), if the Cronbach alpha is less than 0.6, this means that the instrument used has low reliability. If the alpha value is within 0.7, the instrument has acceptable. The internal consistency reliability coefficients (Cronbach’s alpha) for the scales used in this study are all well above the level of 0.7, acceptable for the analysis purpose (Sekaran, 2005).

## Results

As can be seen in Table 1, majority respondents were female (98.2%), had a diploma in nursing (80.1%) and were married (77.9%). The majority were Malay (94.3%), while the rest were Chinese (1.2%), Indian (3.8%) and others (1.1%). The respondents’ mean age was 35 years. The mean for the general nursing experience was 11.41 years while oncology nursing experience was 4.93 years.

Data analysis revealed that cancer care nurses in Malaysia have 13 competencies; (i) empathy, (ii) caring and supportive, (iii) cultural/religious sensitivity, (iv) verbal communication, (v) non-verbal communication, (vi) informing patient, (vii) educating patient, (viii) informing patient family, (ix) educating patient family, (x) drugs administration, (xi) referral, (xii) palliative care, and (xiii) planning. These competencies derive from qualitative findings. Four focus group discussions (FGDs) were held with 17 nurses from three public hospitals. These competencies were grouped into three components; (i) personal (empathy, caring and supportive, and cultural/religious sensitivity), (ii) interpersonal (verbal communication, non-verbal communication, educating patient, informing patient, informing patient family, and educating patient family), and (iii) technical (drug administration, referral, palliative care, and planning). The results showed that personal competency was the highest (4.28), followed by interpersonal (4.10), and technical (4.01) (Table 2).

There are six personal values of cancer care nurses; (i) Do the best job based on religious perspective, (ii) patient satisfaction is priority of cancer care nurse, (iii) job as a nurse is meaningful, (iv) confident as a nurse, (v) commitment in job, and (vi) contribution as a nurse is meaningful to patient. The overall mean score for personal value is 4.45 (SD = 0.45). The highest mean score was 4.53 while the lowest was 4.35 (Table 3).

The findings revealed a positive, significant relationship between personal values and competency (r = 0.59, p < 0.01). (Table 4). Work experience had a positive significant relationship with competency (*r* = 0.113, p < 0.05) (Table 4)

Table 5 shows that only personal values factor was significant in predicting competency. Beta values showed that personal value had a strong predictive power (ß = 0.58, p < 0.01). Personal values) accounted for 34.3% variability in nurses’ competency. 

**Table 1:** Profile of Respondents (N=845)

	Profile	N (%)	Mean	SD
Gender				
	Female	830 (98.2)		
	Male	15 (1.8)		
Age (years)			34.55	10.085
Educational Level				
	SPM/STPM	140 (16.6)		
	Diploma	677 (80.1)		
	Degree	21 (2.5)		
	Master	3 (0.4)		
	Other	4 (0.5)		
Marital Status				
	Married	658 (77.9)		
	Single	179 (21.2)		
	Divorced	8 (0.9)		
Ethnic				
	Malay	797 (94.3)		
	Chinese	10 (1.2)		
	Indian	29 (3.4)		
	Others	9 (1.1)		
Nursing Experiences			11.41	9.291
Oncology Nursing Experience			4.93	4.054

**Table 2:** Mean by Competency Type

	Mean	Std Deviation
Personal	4.28	0.38115
Interpersonal	4.1	0.36637
Technical	4.01	0.44578

**Table 3:** Mean Score for Item Personal Values

	Mean	Std. Deviation
From my religious perspective, I should give the best to my job	4.53	0.589
Patient satisfaction is my priority	4.5	0.523
My job is meaningful to me	4.48	0.521
I am confident in my ability as a nurse	4.42	0.531
I have high commitment in my job	4.42	0.548
My contribution as a nurse is very meaningful to my patients	4.35	0.538

**Table 4:** Relationship between Experience, Personal Values and Competency

Variable	*r*	p
Personal values	0.59	0.001*
Experience	0.11	0.047*

*Significant at 0.01 level (2-tailed).

**Table 5:** Multiple Regression Analysis of Factors Predicting Nursing Competency

Variable	Beta	t-value	Sig
Personal values	0.58	20.43	0.00*
Experience	0.05	1.74	0.08*

R, 0.587; F, 212.436; Adjusted R², 0.343

## Discussion

Majority of nurses in Malaysia is women. This finding is consistent with Maslach et al., (2001) that nursing is dominated by women and nursing workforce in Malaysia is almost exclusively for women (Birks et al., 2009). Gjerberg and Kjolsrod (2001) also mentioned that nursing has always been and continues to be a predominantly female occupation. 

Majority respondents have a diploma level of education consistent with Birks et al., (2009) observation that most nurses in Malaysia have diploma level and a certificate. According to Shamsudin (2006), basic nurses’ education in Malaysia is a certificate level before the qualification was upgraded to diploma in 1990. An educational requirement for nursing in Malaysia differs from another country, for example, Thailand, where the entry-level of practice is the baccalaureate level and Canada (Wolf et al., 2010). 

Personal competency includes empathy, being caring and supportive, and cultural/religious sensitivity. The interpersonal competency component includes the ability to communicate between cancer care nurses and patients, educating/informing patients, and educating/informing the patients’ family. Technical competency in this study is about drug administration, referral, palliative care and planning. Generally, all nurses are technically competent in clinical tasks and clinical procedures, such as administering chemotherapy treatment. Empathy is an interpersonal skill to help people (Reynolds and Scott, 1999). Nurses need to become caring and supportive of the patient in order to help them recover (Sanson-Fisher et al., 2000). Positive experiences with people from other cultures motivated nurses to gain a deeper understanding of cancer patients in terms of cultural/religious sensitivity (Huang et al., 2009)

Verbal and non-verbal communication is one of the competencies that nurses in this study should have in delivering their task. These communications (verbal and nonverbal) competency is consistent with Sheppard (1993); Cleary et al., (1998); Pai et al. (1999); Wilma et al., (1999); Larsson and Butterfield (2002); Tzeng and Ketefian (2003); and Rosenfeld et al., (2012). 

Cancer care nurses have to inform and educate patient and their family. Treacy and Mayor (2000) reported that oncology nurse needs to understand the patient’ educational needs in order to inform and educate them. This data is also consistent with Peppercorn et al. (2011), that patient education is an important function in nursing practice. Besides cancer patient; nurses also have to educate patients’ family. Family caregivers play an essential role in caring for cancer patients (Vaartio-Rajalin, 2011). Nurses are primarily responsible for ensuring patients receive chemotherapy safely (Verity et al., 2008). 

Besides, nurses become a referral to a cancer patient. They suggest to the doctor to refer their cancer patient to a cancer support group, cancer survivor, religious/spiritual resources, social welfare, and counsellor if needed. It is consistent with the study reported by Cleary et al., (1998). 

Palliative is one of the competencies of a cancer care nurse. Nurses are able to provide spiritual and emotional support to a cancer patient. They are also able to provide symptom management to patients who are terminally ill. Some cancer patients will require speciality palliative care intervention to overcome their problems (Skilbeck and Payne, 2003). Palliative care is a growing specialty in nursing care (Doyle et al., 2005). 

Cancer care nurse needs to plan and manage their work routine to accomplish their task well. This finding is similarly reported by Rosenfield et al., (2012) that nurses have to plan and organize their work. 

The competencies of Malaysian cancer nurses are quite similar to that from other countries. Cancer care nurses are empathy and caring, and supportive to cancer patients. They also respect the patients’ cultural or religious sensitivity. Since Malaysia is a multicultural country, the culture is diverse. Cancer care nurses communicate with patients both verbally and non-verbally. They communicate on the disease, treatments, and side effect of chemotherapy. 

Patient education is important to cancer patients. One of cancer care nurse competency is to provide relevant information to them. Cancer care nurses also have to be competence in drug administration (such as chemotherapy procedure). They also have to plan their work effectively. 

From the personal value context, majority respondents agreed that working as a nurse is meaningful to them. From their religious perspective, they believe in giving their best to patient. High majority of respondents are Malay (Muslim). However, the noble value to give their best cuts across ethnicity and religion. This data is consistent with Shahriari et al., (2012) values are shaped by cultural environment, social groups, social systems, religion and past to present experiences. This is the highest value of this study (4.53). 

The belief that excellent service should be given to patient is also consistent with Chen et al., (2008). The findings also show that the nurses are confident in their work ability. They are passionate and give high commitment to their job. Patient satisfaction is important to them. Their jobs and contribution are meaningful. These positive values influence their job commitment, supporting Tzeng (2002) study. 

The findings revealed a positive, significant relationship between personal value and competency of cancer care nurses (r = 0.59, p<0.01). It shows that when the personal value increases, competency increases too. This finding supported previous studies by Altun, (2000) and Horton et al., (2007). According to them, personal values can reflect the nurses’ attitudes and influence professional lifestyle. Horton et al., (2007) stated that to become a good nurse, the nurse herself/himself need to understand her/his value. Cancer care nurses should have a high personal value in order to become good and competent cancer care nurse. 

Nurse experience has a significant relationship with competency. The more experience they are, the more competent they become. The findings supported earlier work by Humpel and Caputi, (2001); Meretoja et al., (2004); Pitayavatanachai (2005); Salonen et al., (2007), and O’leary (2012). Cancer care nurses who have more work experience are more confident with their task. An experienced cancer care nurse makes a patient more comfortable, the maturity that comes with age and life experience can enhance professional nursing competence (O’Leary, 2012). The longer the experience, the greater their self-assessed level of competence. 

The finding indicates that personal value is the main contributor to cancer care nursing competency. The personal value variable explains 34.3% of the total variance in nursing competency. The other 66% of the variability can be explained by other factors which were not explored in this study. Personal value was the strongest predictor of cancer care nursing competency (ß = 0.58, p < 0.01). This showed that cancer care nurses with high positive personal value are more competent. 

Cancer care nurses in 38 public hospitals in Peninsular Malaysia mainly comprised of women with diploma qualification. Competency of cancer nurses in Malaysia is similar to other nurses worldwide. The cancer care nurses studied have 3 domains of competency; personal, interpersonal. and technical. This competency includes empathy, caring and supportive, cultural/ religious sensitivity, communication (verbal and nonverbal), informing/ educating patients and their family, drugs administration, referral, palliative care, and planning. Malaysian cancer care nurses have a high personal value. Personal value is significantly related to cancer care nurses’ competency. Cancer care nurses who have high personal value are more competent. Work experience is significantly related to cancer care nurses’ competency. The more experience the cancer care nurses, the more competent they become. 

Cancer care nurses with high personal value are more competent. Hence look for these criteria in the nurses’ recruitment. Work experience is significantly related to cancer care nurses’ competency. Thus, opportunities should be given to those with inadequate oncology work experience to acquire the learning experience, formally or in-formally. 
